# Local knowledge in inclusive education policies in Africa: informing sustainable outcomes

**DOI:** 10.4102/ajod.v11i0.941

**Published:** 2022-01-31

**Authors:** Chioma O. Ohajunwa

**Affiliations:** 1 Department of Global Health, Faculty of Medicine and Health Sciences, Stellenbosch University, Cape Town, South Africa

**Keywords:** inclusive education, Africa, local knowledge, policy, community, inclusion, schooling, sustainable

## Abstract

**Background:**

This article presents on the outcomes of a study that focused on an analysis of inclusive education (IE) policies in South Africa, Ghana and Uganda. Persons with disabilities live within communities and are raised by the values that apply within their communal context. Policymaking is intricately linked to policy implementation, and the inclusion of local knowledge strengthens policy influence, impacting on implementation processes.

**Objectives:**

This research study explored the definition and foci of inclusion, whether local knowledge is included and how it is represented within the national inclusive education policy in South Africa, Ghana and Uganda. This study reports on the outcomes of the second objective on inclusion of local knowledge.

**Method:**

A qualitative, critical, interpretative and constructivist approach was utilised for the study. Data were gathered through a desktop review and in-depth, individual interviews.

**Results:**

There is inclusion of some local knowledge within the national policies; however, this is minimal and insufficient. Participants argue that even when it is included, it is often embedded or implied, that local knowledge should be made more prominent within inclusive education policies as local knowledge is a community resource that supports policy implementation.

**Conclusion:**

The inclusion of local community knowledge and ways of knowing within inclusive education policies is viewed as a critical and an integral aspect of policymaking. It will help to address the challenges of stigma and negative attitudes, promoting a continuity of knowledge that supports local values and well-being of children with disabilities and their communities.

## Introduction

There is an established connection between education and health, as education is a social determinant of health (Albert & Davia 2011; Fatima 2011; Ross & Wu 1995). The attainment of high levels of education directly enhances a person’s state of health and well-being positively. Studies conducted in Europe, Africa and other parts of the world have revealed a direct impact of the educational level of a person on his or her capacity to access economic and social resources that affect their quality of life (Jude, Houeninvo & Sossou 2015; Shankar et al. 2013; Telfair & Shelton 2012).

Ross and Wu (1995), proposed three key areas where education also indirectly has an impact on our health and well-being - work and economic conditions, social-psychological resources and health lifestyle. Whilst not excluding the psychosocial impact of work and economic resources of income, the attainment of education is the most critical socioeconomic status related to health (Ross & Wu 1995).

Education and health are crucial factors to human capital development (Appleton 2000), which shape opportunities and lived experiences of individuals, locating people within a trajectory in life. Education is a precursor to accessing our socioeconomic rights and should empower and support communities to participate as citizens (Underwood 2005–2006). The psychosocial issues of self-worth and identity formation and such related concepts are also impacted by this experience and positioning. Therefore, education impacts our quality of life.

The quality of life of persons with disabilities (PWDs) is equally impacted by their access or lack of access to education, and PWDs have historically experienced high levels of health disparities when compared with the general population (Krahn, Walker & Correa-De-Araujo 2015). The World Report on Disability (United Nations 2011) estimated 93–150 million children with disabilities globally. Of these children, 6.4% reside in Africa, with less than 10% of all children with disabilities under the age of 14 attending schools. This is a dire situation, as the resultant effect of exclusion in education and consequently economic resources exacerbate poverty and have far-reaching impact on countries in Africa (Kickbusch 2001).

This challenge is beginning to gain the attention of policymakers as evidence points to the link between education, civic participation, and overall well-being. The United Nations Convention on the Rights of PWD situates the educational sector as a fundamental area to explore for the full participation of PWD (UNCRPD 2006).

Sustainable Development Goal (SDG)-4 on education refers to values of equity, inclusion, diversity, equal opportunity and non-discrimination, positing education as a tool for building a moral and just society (Engsig & Johnstone 2015; Magnússon, Göransson & Lindqvist 2019; UNESCO 2020). It is concerned with issues of diversity, equity and the building of a moral and just society as stipulated by the SDG above that we argue for the relevance of including local or citizen-generated knowledge into policy development.

The policy document is a space of politics, contested values and negotiations (Rata 2014). The language, text and focus of the policy document reflect ideological contestations that showcase government intentions regarding any sector (Nugroho, Carden & Antlov 2018). Policy documents do not exist in a vacuum, and they are informed by existing policies and/or context. Policies are influenced by different ideologies that may portray opposing objectives and reflect the understanding and definition given to the focus of the policy. It equally reflects political intent, aim, priorities and strategies for implementation of its focus (Canagarajah 2002).

Government policies frequently straddle different knowledge paradigms. The one paradigm is linked to the global capitalist political economy, whilst the other relates to the country’s own ideals and identity that the nation-state enshrines within its constitutions (Rata 2014). These ideals in the constitution often emanate and are equally influenced by the push for the achievement of democracy and equity within the country’s citizenry. Therefore, the country tries to balance the interests of global forces that it must gratify to some extent, and at the same time, both serves and perpetuates its own ideals within its citizenry (Rata 2014). Education is the place where these ideals are negotiated.

Even within inclusive education (IE), there are different understandings and interpretations, which indicate different value systems and prevailing understandings within specific contexts (Holmes & Crossley 2004; Jones 2009; Magnússon et al. 2019). These challenges are evident within the policy document and context, and different actors in the development of the policy document are likely to be in favour of different ideologies. Therefore, there is no concerted agreement on one singular definition of IE because the understanding given to the concept is influenced by cultures and contexts. Inclusive education is traditionally about education for all (UNICEF 2012). Inclusive education is defined in this study as embodying philosophical frameworks and distinct understandings of the purpose of education. This is more than a set of strategies for educating learners; however, it includes attitudes, values and beliefs that go beyond the school to include the wider community and their local ways of understanding the world around them.

Local knowledge is defined here as a ‘cultural system which becomes common sense for people who share a communal sensibility’ (Geertz 1983). Local knowledge is positioned as more sensitive to local realities (Boossabong 2017; Smalley 2020), which can support implementation more than the direct importation of foreign and global ideologies that are presented as scientific facts but might not be relevant or suitable for local realities.

Inclusive education is about belonging, membership and acceptance (Singh 2009 cited in Ciyer 2010). Therefore, the exclusion of or insufficient inclusion of local knowledge within the IE policy belies the statement given above. The inclusion of local knowledge as a policy mandate supports a continuity of learning for the child across the school and home contexts, and acknowledges the partnership of the home, school and community in the education of a child as given within IE principles.

Therefore, this study explored whether local knowledge is included and how it is represented within IE policies in South Africa, Ghana and Uganda.

## Theoretical framework

This research study is situated within post-positivism (Fischer 2002; Fuller 2009) and informed by a critical, interpretative, constructive paradigm (Boossabong 2017; Boossabong & Chamchong 2019) within a social justice framework, as is the tenets of IE which speak to equal participation, equity, non-discrimination and social justice.

The critical policy analysis advocates for the inclusion of local knowledge in policymaking, emphasising the value that local knowledge brings to policy development, and the interpretive, constructivist analysis framework encourages the merging of various kinds of knowledge in policymaking and asserts that knowledge is pluralistic. This framework further hypothesises that the technocratic approach that has been normalised within policymaking contributes to a generic approach to policymaking (Boossabong 2017) that takes inadequate consideration of local, contextual socio-cultural knowledge, and advocates for the inclusion of varied knowledge in policy development. Therefore, within constructivism we are not excluding other forms of knowledge, but arguing for a holistic approach that includes and represents different kinds of knowledge, especially contextually relevant knowledge (Ohajunwa 2019).

## Study methodology

A qualitative, critical, interpretative and constructivist approach was utilised for the study, as aligned to the postpositivist values that inform this study (Boossabong 2017; Boossabong & Chamchong 2019).

Data gathering was performed in two phases. The first phase is a document review and analysis of national government IE policy and government statements according to the UNESCO Global Education Monitoring Report (2020) priorities for national policies. However, this study is focused on an outcome from the second phase, related to local knowledge inclusion within IE policies in the three contexts chosen for the study.

The second phase of data gathering was carried out by conducting interviews and/or storytelling (Chilisa 2012; Easby 2016) to elicit historical narratives of place and context as aligned to the critical approach (Ohajunwa 2019). Focus group discussions were initially included but had to be removed because of the COVID-19 pandemic, as people could not travel to spaces where they could meet as a collective. There was an attempt to have an online focus group discussion; however, participants could only go online at different times, and some struggled with connectivity within their context. Data were gathered through telephonic one-on-one interviews and WhatsApp calls (Lo Iacono, Symonds & Brown 2016). Zoom and Microsoft Teams were also used, according to participants’ preferences (Archibald et al. 2019). The platform supported confidentiality and protection of participant information, as noted by researchers and participants who have used Zoom as an interview platform (Archibald et al. 2019). The data were secured and saved in the clouds with a password known only to the researcher.

The study methodology aligns with the postpositivist philosophy of ensuring multiple sources of data from various participants to gain a holistic sense of the phenomena, as knowledge is positioned here as socially constructed.

## Study setting

The study setting included South Africa, Ghana and Uganda, which were from three different regions of Africa (Southern, Western and Eastern) were intentionally chosen for representation. Therefore, individuals from these three countries participated in this study.

## Study sample

Purposeful sampling was carried out, and through snowballing, potential participants were identified (Creswell 2013) by approaching and collaborating with the African Network for Evidence-to-Action on Disability (AfriNEAD) country coordinators.

AfriNEAD is a flagship project of the Centre for Disability and Rehabilitation Studies at Stellenbosch University. AfriNEAD supports the much-needed translation of research into evidence-based advocacy, practice and policy, particularly in the pan-African context by facilitating dialogue across stakeholders through their network of disabled people’s organisations (DPOs), non-governmental organisations (NGOs), and various community-based practitioners, policymakers and academics (Ohajunwa et al. 2017). With this reach, the AfriNEAD country coordinators were able to direct the researcher to DPOs, policymakers and other persons of interest who could give rich information regarding the study focus.

The NGOs or DPOs selected needed to be involved in at least one or all the areas below:

- The promotion of access to education for children with disabilities
- Collaborations with schools and teachers aimed at supporting the successful implementation of IE
- Engagement and advocacy with parent organisations or communities on the relevance of IE and policies
- Government advocacy on IE and policies.

A thematic analysis of data sets was carried out manually, and the findings related to the theme on inclusion of local knowledge are presented below. Each data set was analysed manually, and units of meaning that speak to the research question were identified. These units of meaning were then colour coded, copied and put on an excel spreadsheet in separate columns for each transcript. The units of meaning with similar colours were further analysed and given a code; this was performed first for each transcript, and then across all the transcripts. As cross analysis occurred, all codes were put into categories, and outliers were identified, and new categories created as needed; this was ongoing until all codes were categorised. The final themes emerged from the categories. During data analysis, the units of understanding that cut across and aligned both the document review and interview analysis are given below:

- understanding of inclusion
- the foci of inclusion
- identification of historical narrative and contextual knowledge
- local knowledge inclusion and how it is represented within the national education policy and government statements on IE.

Table 1 shows the participants in this study across the three countries.

**TABLE 1:** The study sample according to the country.

Country	Affiliation	Number
South Africa	AfriNEAD country coordinator	1
	Higher education/institute	3
	NGO/DPO/PWD	5
	Inclusive education specialist/institute	1
	Policymakers	0
	Teachers	0
Ghana	AfriNEAD country coordinator	0
	Higher education/institute	0
	NGO/DPO/PWD	1
	Inclusive education specialist/institute	1
	Policymakers	3
	Teachers	2
Uganda	AfriNEAD country coordinator	1
	Higher education/institute	1
	NGO/DPO/PWD	4
	Inclusive education specialist/institute	0
	Policymakers	2
	Teachers	0

	**Total**	***n* = 25**

AfriNEAD, the African Network for Evidence-to-Action on Disability; PWD, persons with disabilities; DPO, disabled people’s organisations; NGO, non-governmental organisations.

## Findings

All participants (*n* = 25) agreed that it is very important to include local community knowledge within the policy document. They also reiterated the relevance of including local knowledge and ways of understanding to inform policy processes:

‘Absolutely vital! You cannot have it without community input, local knowledge. Otherwise, it’s not going to be applicable to the teachers, to the learners, to the community if you don’t have that. We’ve learnt that this doesn’t work. If you import things from other countries that are not applicable to our context, it’s going to fail. It’s not relevant. So, absolutely, it’s vital to have local community knowledge. It’s from the resources we use, the images, the pictures we have in our workbooks for our kids, it’s the examples that are used. If those are relevant and applicable… Your Janet and John work readings and books, I mean [*laughs*]. It is scary.’ (SA P1, Inclusive education practitioner, higher education, 12 February 2021)

‘As long as we want to address the local problems of our people, it means definitely, their perspective of things should also be addressed or understood, understood first, then we have to find a way of addressing them. Because sometimes, diagnosis of problems can be different from different angle. Because you have not diagnosed the correct problems, sometimes you can only diagnose the correct problems by coincidence. But it is better that you actually seek out the views and the perspectives of these people, if a policy is going to address their problems.’ (UG P4, Person with disability, NGO and community practitioner, 21 February 2021)

‘It’s [*local or indigenous knowledge*] very, very important, this is what is missing. To me, if you ask, this is the missing link. Between the numerous policies we have vis a vis, the indigenous knowledge, you know, everything we do in life must have a root and the root must be based on our belief systems. [*Policymakers*] must look for the traditional knowledge, look for the way we live, the way we do our things. Right? Then our way of life could be translated into the policies. So that when we are able to achieve this, it doesn’t become something like an imposition, something created somewhere else, then it’s been imposed on you.’ (GH P6, Person with a disability, higher education, 24 March 2021)

Some participants felt that certain local knowledge are embedded with their national IE policy; however, they agreed that it is insufficient and should be more emphasised:

‘I think I would say there is definitely a space for community knowledge in IE. I mean, if I look at our current policy, we have a very Western view of the child in our policy. Ideas from the UK, Canada, and probably in the US, I think, obviously, maybe Australia, there’s very little of Africa, you know, when we use the word Ubuntu and that is that. Okay, cover that box. We covered Africa, we used Ubuntu.’ (SA P9, Inclusive education practitioner, 15 March 2021)

‘Indigenous knowledge should be included when fully formulating policies like I said, you cannot underrate indigenous knowledge. Which I must say that this is a policy we have currently, even though we have few of their representatives, but that is not enough.’ (GH P4, Policy maker, 27 April 2021)

‘Policy being a small thing which does not talk about everything, but in some way, there are some of those statements where local knowledge is embedded. Because when we talk about attitude change at local, at the family level so that your experience can accept the child and be with the siblings – because all those things are what are in our curriculum. So I think some of those things are embedded, although somebody may not be able to see them very straightforward. We are talking about local knowledge here, this is how the policy is going, but later on when we unpack those strategies, I think that we have embedded them because it is very important local knowledge.’ (UG P6, Policy maker, 06 march 2021)

There was an impression that policymakers can be far removed from the realities of the citizenry and communities on ground:

‘Like I told you earlier, if you look at the reasons as to why certain children are not actually going to school, these are not reasons which you can sit in an office and think about sometimes you have to actually go down there and look at them. Then triangulate your information and look at the kind of design your policy is going to take the kind of shape it will take. So, I definitely think that local knowledge should actually be the first thing we actually say incorporating our policy is going to actually address problems of those local people, because we are not designing policies to work for us at the top. No, we are designing policies that should work to change communities and the ground at the bottom.’ (UG P4, Person with disability, NGO and community practitioner, 21 February 2021)

‘It should be central. So it’s the same as saying that learner voice should be central. It’s the whole idea of you can’t make policies for people without including people in the policymaking process. It’s essential for participatory democracy. So, learners, communities, should be an integral part of deciding how they want to live, and how they want to be governed and how they want policies to be developed…so then it becomes in my view, government obligation to realise that that’s what the people want.’ (SA P10, Inclusive education policy analyst, 15 February 2021)

Participants provided various reasons why it is important to include local knowledge, stating that the implications can be far-reaching, influencing even the curriculum, school and community. They insisted that including local knowledge would support more buy in from the community and support a contextually relevant and diverse curriculum for children with disabilities:

‘But also the school can learn a lot from the home environment activities in the home, how the home communicates with this deaf blind child, etc. So we have taken care of this - I hope that when the document comes out of cabinet that they maintained this. All this comes out from considering the kind of culture the people in a particular area have, the kind of practices, what is possible to include in the school curriculum, how the curriculum can cater for their way of life.’ (UG P7, Person with a disability, higher education, 25 January 2021)

‘So, we basically mean, so for me, that would be and I would really include children’s knowledge in community knowledge, you know, what’s happening in that particular community? What happens in that way that child lives? What are the elders saying? And the elders can be the grandmothers or the neighbours, you know, what are your neighbours saying? What, what’s allowed? What isn’t allowed? Well, you know, what’s the issue around violence? You know, we think children are not aware that violence is happening around them, they are aware. Yeah. And to me, there’s a lot of we’re not allowing that that information. And it’s not information, it’s embodied knowledge, almost, it’s embodied, it’s part of who they are. We kind of leave that at the door at school, like, Okay, can you leave that there now and come inside? And that’s problematic.’ (SA P9, Inclusive education practitioner, 15 March 2021)

‘Community knowledge for me is the credible source we have to actually rely on when it comes to changing attitude, changing characters, changing systems, because for me, when you involve the community from the beginning, don’t think for them, let them bring out the issue and let them bring out the solutions. So, when they bring out the issues, they bring out the solution like I indicated, then they are they are now they’re watchmen or the security men should protect what they want to realize.’ (GH P4, Policy maker, 27 April 2021)

Participants referred to the challenge of the negative stereotyping of and attitudes to disability within some African communities as problematic:

‘It’s such a difficult one because all we have known in this country is segregation. Our whole history has been a history of segregation. And then we also have the added complexity of a lot of traditional knowledge of disability in particular being sort of routed in… sort of cultural traditions of understanding disabilities as curses and… so you know.’ (SA P10, Inclusive education policy analyst, 15 February 2021)

‘We will need to work more with the teachers and continue with the sensitization because as you know as Africans disability issues we don’t like it. These are the negative side we don’t want it near us. So, it is always difficult for people to terms.’ (GH P2, Policy maker, 17 February 2021)

‘And when they come out of their house where do they pass? They’re passing through the community. Has the community really prepared that path or that road? The people they are meeting, if they are going to say “oh look at that hooligan,” “look at that moron,” if they use that language… transiting from his home to school and people are naming mean names, they’re beating me, throwing stones on me because I am either disabled or albino or whatever, then it will interfere with my learning. Then after school, out to the outside. So it is very important, local knowledge, local understanding or whatever plays a big role.’ (UG P6, Policy Maker, 06 March 2021)

However, they believed that despite these challenges, including local knowledge could only inform these challenges of stigma and would make policy implementation more achievable on ground:

‘This kind of local knowledge should actually be incorporated in the policy. And I think it will go a long way, in terms of facilitating education, IE for our children, because the problems you’re dealing with actually, coming from down there, those negative perceptions about why certain children may not be able to go to school, I think they should be incorporated [*Addressed*]. But at the same time, when you talk about the local knowledge, it still goes down to even the other players, like the teachers, because I take teachers will also be from, from the local the localities, in our communities, what are the issues regarding how they’re able to teach these children.’ (UG P4, Person with disability, NGO and community practitioner, 21 February 2021)

‘Within our beliefs, and then what they believe in then, we include them in the whole policymaking. They [*the community*] feel part of it, and therefore, they will be able to help us implement this, because we have also considered them.’ (GH P1, Inclusive school educator, 23 March 2021)

‘I just don’t think it will assist, I think it is essential [*including local knowledge*]. I think like really it is a huge gap. Say for example, children with disabilities. We know that there are negative sides of it. Lots of stigma and discrimination around children with disabilities. And that can affect children actually coming to the school and being part of the school because the parents don’t actually feel like they can bring the child to the school. So that is a negative impact of local knowledge and if one understands that, then one would be better able to do that.’ (SA P7, Inclusive education practitioner, higher education, 12 February 2021)

‘There is an education that gives you skills to be able to get a living, sustain a living income, but there is an education for life that starts from the home, where your, your attitudes to life, your worldview is shaped by your parents, and the education at home. And the school can only build on the foundation of what your parents have laid in your life. Yes, a crab doesn’t give birth to a bat.’ (GH P7, Inclusive school educator, 24 March 2021)

In Uganda, participants confirmed that a stronger focus on the link between a school and home has been more emphasised within their current IE national policy, and all participants stated that there was wide consultation regarding IE policies in their respective countries. Local communities were often represented through DPOs, NGOs, traditional leadership and religious leaders; however, still the local realities differ from policy directives:

‘One of the areas where reform emphasis has taken place in the national curriculum for education in Uganda is use of indigenous cultural practices. In the process of developing the current draft of the special needs and IE policy, we made emphasis on participation of parents. So, we have emphasised the use of home-school partnerships in this policy.’ (UG P7, Person with a disability, higher education, 25 January 2021)

## Discussion

### Value placement on local knowledge

Three types of knowledge influence policymaking, and this knowledge is not mutually exclusive as they often co-exist to varying degrees within the policy processes - scientific knowledge (experimental, quasi-experimental, ethnographic and case study), professional knowledge (bureaucratic, intermediary and activist) and local knowledge (citizen, religious, cultural and experiential) (Nugroho et al. 2018).

Knowledge is ranked according to certain categories. These are knowledge types: institutional arrangement of knowledge, methods in knowledge creation and forms of knowledge, local knowledge is always ranked lowest in comparison with other types of knowledge (Nugroho et al. 2018). Therefore, local knowledge is very often the least supported within the knowledge-to-policy realm, although it is critical as the knowledge ‘on ground’, which is traditionally the space for the experience of the gaps in policy implementation.

Local knowledge, also referred to as citizen knowledge (Jones et al. 2013) or experiential knowledge, is concerned with the same issues as scholarly research but utilises a different lens and meaning making that are informed by context and human engagement with their context to approach policymaking.

### Challenges to inclusion of local knowledge

Some challenges to the inclusion of local knowledge, however, are that- local knowledge is not easily generalisable as it is usually grounded in the context, while public policy would aim to address the needs of the general populace. Local knowledge is also tacit knowledge, and this attribute has been cited as a reason for marginalising local knowledge. This speaks to the value placement on other ways of knowing within policy formation.

The importance attached to IE, its guiding policies and the right of every child to education have grown, and most countries have adopted and created laws and policies to this effect (UNICEF 2012). However, the quality and applicability of these policies differ from between countries (Hayes & Bulat 2017), as every country will approach inclusive educational reforms as influenced by their current educational systems, needs and cultural contexts. This is because the meaning of IE within each country is subject to the priorities set by local policy actors, which are evidenced within the policy directives (Magnússon et al. 2019). Participants in this study express inadequate inclusion of local knowledge as an indication that it is not a government priority.

Evidence from the literature, however, agree with participants’ assertion that the inclusion of local, contextual knowledge will fill a gap. Hayes and Bulat (2017) stated that the ratification or localisation of international policies supports successful implementation. However, the way localisation, inclusion, and/or influence of local or social knowledge within policymaking (Ciyer 2010; Holmes & Crossley 2004; Nugroho et al. 2018; Rata 2014) occurs is influenced by the country’s history and context (Ciyer 2010). This reflects the ways that international and local knowledge patterns respond to global trends and discourses (Openshaw 2009). This influence of history and context is evident in South Africa, where participants often referred to their racialised history and its current impact on the inclusion of diverse knowledge within their IE policy. This remains a challenge within the policy landscape.

### Relevance of including local knowledge within policies

Despite the above challenges, all participants in this study recognised the relevance of including local knowledge within policymaking. The importance and argument for including local knowledge within policymaking has been demonstrated in certain practices within policymaking (Fischer 2002; Magnússon et al. 2019; Smalley 2020), demonstrating the legitimacy of local knowledge to strengthen and influence policy. UNESCO (2020) posited that inclusion cannot be enforced but should be informed by meaningful collaboration between the government and the communities they serve. Participants felt that this sense of authentic collaboration is still missing, as PWDs and their communities are still under-represented within IE policymaking.

The inclusion of local knowledge into policies contribute to globally aware but locally relevant education policies (Boossabong 2017; Nugroho et al. 2018). If we aim to achieve long-term, sustainable goals, there is a need to ensure that local knowledge and cultural values inform policy development in Africa. This alludes to whose knowledge and values are being prioritised within educational policies in Africa? What does this mean for IE and PWD in Africa?

Although international instruments related to the field of education (UNESCO 2020; United Nations 2006) advocate that the understanding and implementation of educational policies should be influenced and grounded in the political, social and cultural philosophies of the local context, the reverse has often been the case with many countries in the global South, whose policies are still being guided mainly by outsider influences (Holmes & Crossley 2004). Africa has an ecology of knowledge that can influence policy development within the continent. Thus, an integration of knowledge is required for development, and ‘traditional wisdom should also be considered as a valuable part of the knowledge system’ (Cetto et al. 1996:27).

It becomes relevant that the ‘knowledge system’ in the context within which the IE policy is developed should influence what is foregrounded within the policy document and what is excluded (Magnússon et al. 2019). Policy, therefore, is ‘in the state of becoming, continually contested and interpreted by those initiating it, by those supposed to implement it and by external actors’ (Magnússon 2015, cited in Magnússon et al. 2019:68). Policy documents are influenced by ideologies, and policies influence practice (Taylor 1997). Inclusive education is not different. The understanding provided to the term ‘inclusion’ should be informed by the prevailing contextual influences and contestations.

It is interesting to note that in the current year (2021), all the three national policies within the three countries of this study were due for an evaluation, so the focus elicited critical issues about what participants would like to see revisited in the new policy, inclusion of local knowledge is one of those areas.

I present that the inclusion of local knowledge within national education policies will support a more contextually relevant reality for inclusion of PWDs in the area of education, health and well-being in their local context.

## Strengths and limitations

This study catered to certain strengths and demonstrated some limitations. Firstly, the study is located and contextualised within three countries located within three different regions of Africa. This context is a strength of the study, as it provided some triangulation in terms of data analysis and methodological rigor. Secondly, data was collected via desktop review of national IE policy and government statements. Although not fully reported in this article, (It is being written up in a different article) the outcomes of the desktop study further corroborated the responses of the study participants. Thirdly, key participants with longstanding experience and engagement in the field of IE within their countries, were interviewed in South Africa, Ghana, and Uganda. Some study participants were involved in the crafting of the current national IE policy in their countries and were able to provide very relevant insight into the status of the national IE policy within their contexts.

One of the key limitations of this study is that three main categories of participants could not be accessed in one or more of the study contexts. Traditional rulers could not be accessed in any of the three countries because of the online mode of data collection because of COVID-19 restrictions. The African Network for Evidence-to-Action on Disability coordinators could not physically go to these community and traditional rulers on my behalf, and therefore, this important voice is missing in the study.

As a result of various levels of gatekeeping challenges, I could not access teachers to interview in all the countries; However, I could only access teachers in Ghana. The South African Education Department granted permission to access teachers; however, the school principals I contacted declined and some schools did not respond to me. In Uganda, I was unable to secure permission, so I could not interview teachers. The final important voice that is missing is only in one country - South Africa - which is the voice of policymakers. I was able to access policymakers in Ghana and Uganda; however, in South Africa, this was challenging. After approximately 4 weeks of emails and follow-up with two identified policymakers, I had to give up and conclude the study.

## Implications and recommendations

Participants of this study have reiterated the importance of including local knowledge within IE policy to inform local realities. The three contexts of this study have formed very good written IE policies through wide consultations with partners and collaborators at different levels. However, participants insist that these policies have not yet fully met the goal of inclusion for all, as more knowledge on ground is needed to inform policy formation and fill the gap experienced in policy implementation related to the education of children with disabilities.

This insufficient inclusion of local knowledge also speaks to the way the policy document is popularised, and the spaces of popularisation. Governments should ensure that popularisation plans and strategies are not simply an ‘information’ session, but a sharing session, a conversation, a collaboration and opportunity to learn from their local communities and inform local communities about the intention of the governments regarding the IE policies. As stated earlier, this year, all three countries are scheduled for a policy overhaul, and therefore, these recommendations from this study are timeous and will hopefully inform these processes.

Policymakers need to engage more with local communities during the policy formation, and not only when it is time to implement policies. An authentic, meaningful engagement should be undertaken with communities to inform IE policy, not just as an additional checklist.

## Conclusion

Persons with disabilities and their educators do not exist in isolation but emanate from communities with existing value systems that have an impact on their lived experiences and access to sustainable educational outcomes. The IE policy document can facilitate access and address the implementation gap that is often experienced by policy implementers through the inclusion of relevant local knowledge within the policy document.

Participants of this study across South Africa, Ghana and Uganda reiterate the relevance of local knowledge for informing sustainable policy outcomes that cater to local realities. Rather than imposing western-imported ideals of education, governments must ensure that these local realities inform educational outcomes within the African context, thus contributing to continuity of learning for children with disabilities and policy implementation within African communities.
